# Ureterocalycostomy - final resort in the management of secondary pelvi-ureteric junction obstruction: our experience

**DOI:** 10.1590/S1677-5538.IBJU.2015.0368

**Published:** 2016

**Authors:** Venkat A. Gite, Ayub Karam Nabi Siddiqui, Sachin M. Bote, Saurabh Ramesh Patil, Anita J. Kandi, Jayant V. Nikose

**Affiliations:** 1 Department of Urology, Grant Govt. Medical College & Sir J.J. Group of Hospitals, Mumbai -Mumbai, India;; 2 Department of Surgery, GMC Aurangabad - Aurangabad, India

**Keywords:** Ureter, Multicystic renal dysplasia, bilateral [Supplementary Concept], Pyeloform [Supplementary Concept]

## Abstract

Ureterocalycostomy can be performed in patients in whom desired methods of treating secondary PUJ (Pelvi-Ureteric Junction) obstructions either failed or could not be used.

In our study, one child and two adults in whom one redo-ureterocalycostomy and two ureterocalycostomies were performed for severely scarred PUJ. The causes for secondary PUJ obstruction were post-pyelolithotomy in one case, post-pyeloplasty and ureterocalycostomy for PUJ obstruction in the second patient and the third patient had long upper ureteric stricture post-ureteropyeloplasty due to tuberculosis. In all these cases ureterocalycostomy proved to be salvage/final resort for preserving functional renal unit.

## INTRODUCTION

Although the spectrum of indication for ureterocalycostomy has changed, it is considered an important salvage procedure to bypass extensive peripelvic scarring and provide non-obstructed and dependent drainage (1, 2).

This technique has been used for over 40 years, more frequently for the management of failed pyeloplasty (3), in case of post pyelolithotomy PUJ disruption/scarring and long stricture in upper ureter (specially due to tuberculosis).

## MATERIALS AND METHODS

Three cases of ureterocalycostomy from 2009 to 2015 were reviewed. They included 3 patients (two males and one female): one child 6 years old, one adult male 35 years old and one female 23 years old. Ureterocalycostomy was done in two patients and redo ureterocalycostomy in one patient. Indications were post pyelolithotomy in one case, post pyeloplasty and post-ureterocalycostomy in one case and in one patient post uretero-pyeloplasty for upper ureteric long length stricture due to tuberculosis. Preoperative anatomical/functional assessment was done by nephrostogram, intravenous pylography, retrograde pylography ± diethyle triamine pentaacetic acid (DTPA) renal scan.

**Figure 1:**
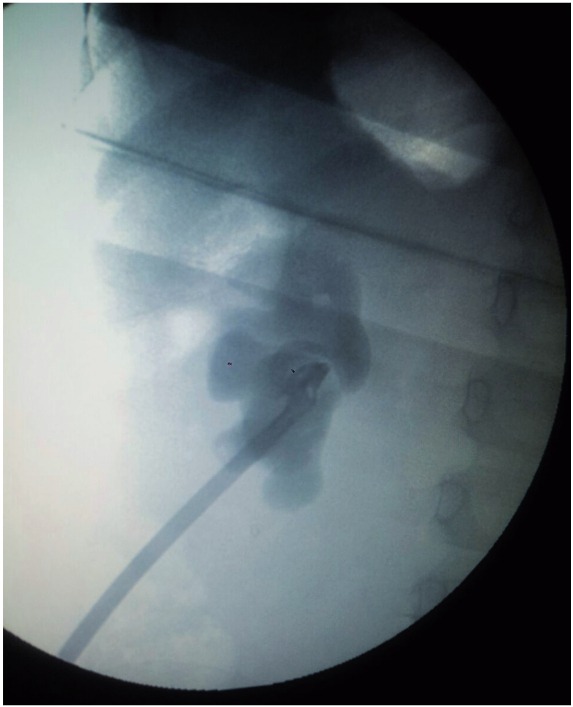
Nephrostomogram showing complete blockage at PUJ.

**Figure 2:**
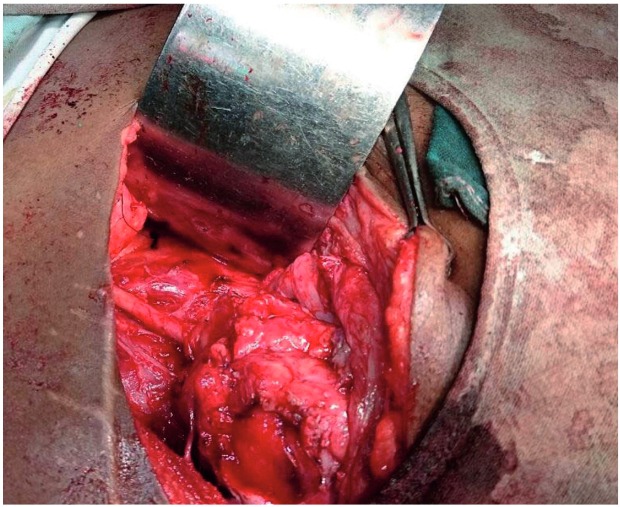
Intraoperative photo–completion of procedure.

**Case 1:** A 5 year-old male child had 1.5cm PUJ calculus with total intrarenal pelvis. He underwent pyelolithotomy in general surgery unit. During the procedure, he had PUJ disruption which was sutured over double J (DJ) stent. Post operative course was uneventful. DJ stent was removed after 6 weeks. Patient developed pain and fever after stent removal. He had progressive hydronephrosis on serial ultrasound (USG) scans. Retrograde Pyelography (RGP) with DJ stenting was tried but guide wire could not be passed beyond PUJ. Then the patient was submitted to ureterorenoscopy (URS) and complete blockage beyond the level of upper ureter was detected. Percutaneous nephrostomy tube (PCN) was placed as drainage procedure and nephrostogram (Figure-1) was done after 2 weeks, which confirmed complete PUJ blockage. Due to the presence of intrarenal pelvis and gross periureteric fibrosis we decided to perform ureterocalycostomy (Figure-2) as first choice which was performed after 6 months from primary procedure.

**Figure 3:**
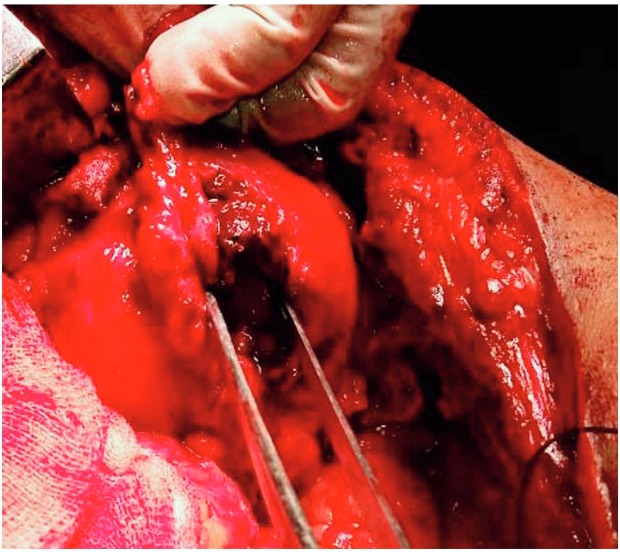
Intraoperative photo-Guillotine technique.

**Case 2:** A 35 year old male underwent left pyeloplasty for left pelviuretric junction obstruction. After DJ removal, at 6 weeks he developed pyonephrosis and perinephric abscess for which percutaneous nephrostomy and drainage of abscess was done. At that time, on evaluation by nephrostogram, DTPA renal scan and retrograde pyelography, he was found to have functioning and obstructed renal unit. On nephrostomogram he had complete obstruction distal to pelviuretric junction. He was submitted to left ureterocalycostomy with DJ and PCN elsewhere. DJ stent was removed subsequently. Patient presented to us 9 months ago with features of pyonephrosis on left side for which he underwent left PCN insertion. Subsequently he was re-evaluated by nephrostomogram, computerised tomogram, intravenous pyelography (CT-IVP), DTPA scan and found to have normal functioning obstructed left renal unit without drainage beyond ureterocalycostomy site. RGP and DJ stenting were tried but failed due to inability to pass the guide wire beyond anastomotic site, suggestive of anastomotic stricture. We performed a redo-ureterocalycostomy by guillotine technique (Figure-3) with DJ stenting. Intraoperatively we found gross perianastomotic site fibrosis and anastomotic site stricture. Contour of lower pole of kidney was maintained suggestive of previous anastomosis by incision/coring technique.

**Figure 4:**
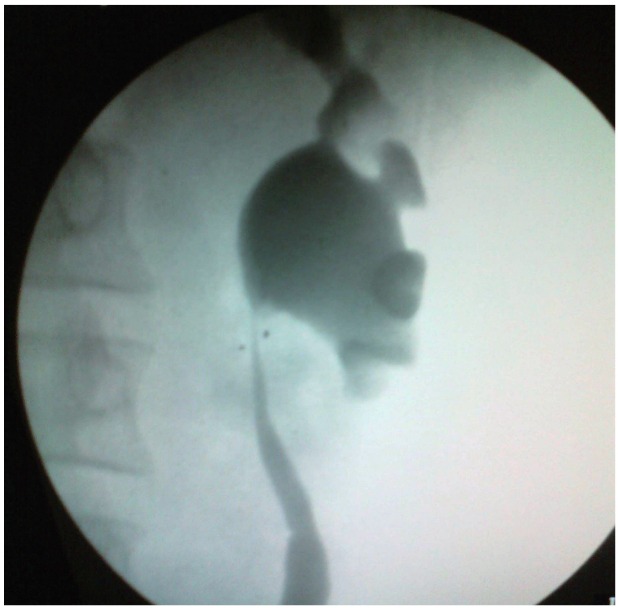
RGP showing left upper ureteric stricture.

**Case 3:** a 23 year old female with left upper ureteric stricture secondary to tuberculosis (proved on urine AFB positive) with solitary functioning kidney. She underwent left DJ stenting and anti-tuberculous treatment. After completion of intensive phase, she underwent left ureteropyeloplasty over DJ with excision of strictured segment elsewhere 4years ago. After DJ removal at 6 weeks, she developed pain, fever and pyonephrosis for which PCN was placed and referred to us. Subsequently, she was evaluated by nephrostogram, CT IVP, and DTPA scan and found to have normal functioning obstructed left renal unit without drainage beyond anastomotic site. RGP (Figure-4) and re- DJ stenting tried but failed due to inability to pass the guide wire beyond anastomotic site suggesting anastomotic stricture. Three years ago, we did ureterocalycostomy by guillotine technique over DJ stent. Intraoperatively she had gross perianastomotic site fibrosis and anastomotic site stricture. After 6weeks, DJ was changed as RGP showed inadequate drainage. Subsequently she needed two more DJ stent changes, after which she had non obstructed drainage which was evident on last RGP-IVP.

## RESULTS

**Figure 5:**
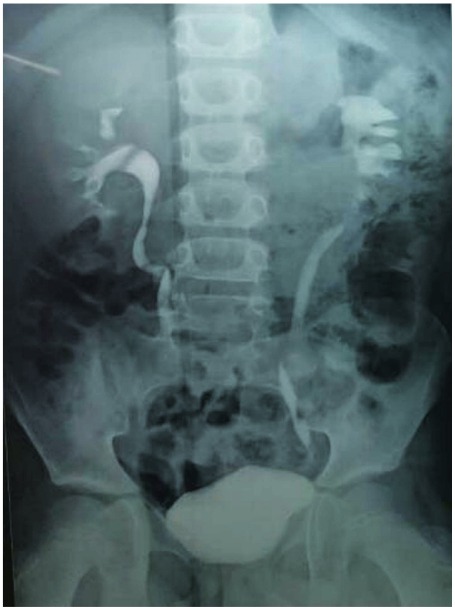
Follow-up IVP-adequate drainage across anastomosis.

Three patients underwent ureterocalycostomy, out of which one had redo-ureterocalycostomy. Demographic profile is shown in Table-1. Two patients underwent primary procedures in other centres and one patient in our centre by general surgery unit before referring to Urology unit. One patient was primarily treated for pelvic stone with total intrarenal pelvis, one for PUJ obstruction and one for left upper ureteric stricture secondary to tuberculosis. Pre operative profile is shown in Table-2. All three patients were asymptomatic till last follow-up (1 case after 3 times change of stent every 3 months) with objective evidence of obstruction relief (Figure-5) (Table-3).

**Table 1:** Demographic profile.

	Case 1	Case 2	Case 3
Age(years)	6	35	23
Gender	Male	Male	Female

**Table 2:** Preoperative and Intraoperative findings.

	Case 1	Case 2	Case 3
Primary disease	Pelvic stone with intrarenal pelvis	PUJ obstruction	Left upper ureteric stricture(TB)
Primary procedure	Pyelolithotomy	Pyeloplasty and Ureterocalycostomy	stricturoplasty
Complications / event in perioperative period during primary procedure	PUJ disruption	Wound infection and long term leak, perinephric abscess	-
Primary procedure	Our centre, by Surgical unit	Other centre	Other centre
Salvage Procedure done	Ureterocalycostomy	Redo-Ureterocalycostomy	Ureterocalycostomy
Intraoperative findings	Severe peripelvic and periureteric scarring+	Severe scarring at perianastomotic site	Severe peripelvic and periureteric scarring

**Table 3:** – Outcome.

	Case 1	Case 2	Case 3
Hospital stay(days)	14	15	14
Follow up	1 year	6 months	3 years
Relief of obstruction	Evident on IVP	Evident on IVP	Evident on IVP after change of stent, 3 times every 3 monthly
Reduction in hydronephrosis on USG	+	+	+

## DISCUSSION

Historically described by K. Neuwirt in 1947 (4), the surgical technique most commonly used today was delineated by Hawthorne et al. (5). Due to its particular indication, this procedure is rarely performed and operator has limited experience (6). Basically it is used for pyeloureteric union in which conventional pyeloplasty cannot be performed, whether as first treatment or repeated surgery, due to complications of kidney stone surgery or for the treatment of renal or ureteric complications of tuberculosis (7) The most frequent indication is scarring resulting from previous open surgery for stone removal or repair of PUJ obstruction (8). In many cases redo-pyeloplasty or endopyelotomy are the alternatives which can be used (9). In our cases, these options could not be used as in one case, pyelolithotomy was done for PUJ stone with total intra-renal pelvis having intraoperative disruption of PUJ, in the second case pyeloplasty and once ureterocalycostomy and in the last case due to post-ureteropyeloplasty for long upper ureteric stricture. Secondary PUJ stenosis following conventional pyeloplasty, open stone surgeries or endopyelotomy is complicated by peripelvic inflammation and dense fibrosis caused by urine extravasation which make them unsuitable for redo PUJ reconstruction (10).

Preoperative patient evaluation is the key to success for the procedure. It is imperative the location and the extent of the disease segment assessed with preoperative imaging including retrograde/antegrade pyelography/IVP and nuclear renography to assess the renal function (1). Surgical techniques used in all cases include some key points which are access through virgin area, dissection of ureter with good amount of adventitial tissue, guillotine amputation of lower pole parenchyma, wrapping of anastomosis with omental graft, stenting of anastomosis with proximal diversion and achieving additional length by renal descensus, resection of ureteric tissue until normal and vascular tissue is identified with wide lateral spatulation (10). Guillotine amputation of lower pole is better than simple wedge resection or incision technique to avoid the anastomotic stricture (11). As in our second case, in which previous surgeon used incision/wedge resection technique during redo-ureterocalycostomy, contour of lower pole of the kidney was found intact.

In genitourinary tuberculosis, long upper ureteric stricture may develop. For such cases, Couvelaire reported good result with ureterocalycostomy (12). We had one case who was treated with antitubercular treatment with double J stent and after 6 weeks, ureteropyeloplasty was done elsewhere which subsequently failed for which we have done ureterocalycostomy with renal descensus and nephropexy. Same patient needed three times change of stent every 3 months to achieve non-obstructed drainage evident on IVP.

Applying technical details meticulously leads to better results. No patient had major complication except one with proven genitourinary tuberculosis who needed three times change of stent which may be due to minimal residual disease which got settled later on.

Post operative results were assessed by USG/IVP with or without isotope renal scan in all cases and had resolving hydronephrosis with non-obstructive drainage with minimum follow-up of 6 months and maximum of 3 years. Shah TP et al. operated 25 cases of ureterocalycostomy of which 22 had clinical and radiological improvement (10). Arap et al. reported clinical and radiological improvement after laparoscopic ureterocalycostomy for complicated upper urinary tract obstruction in all 6 cases after median follow-up of 30 months (13).

## CONCLUSIONS

Ureterocalycostomy is the final resort for salvaging functioning renal unit having complex secondary PUJ strictures. Likely situations include post pyelolithotomy for PUJ stone with complete intrarenal pelvis and PUJ disruption, long upper ureteric stricture (TB) and post ureterocalycostomy anastomotic stricture, if it is done by incision/wedge resection technique primarily.

## References

[1] Matlaga BR, Shah OD, Singh D, Streem SB, Assimos DG (2005). Ureterocalicostomy: a contemporary experience. Urology.

[2] Mesrobian HG, Kelalis PP (1989). Ureterocalicostomy: indications and results in 21 patients. J Urol.

[3] Selli C, Rizzo M, Moroni F, Dedola G, Amorosi A (1992). Ureterocalicostomy in the treatment of pyeloplasty failures. Urol Int.

[4] Neuwirt K (1947). Implantation of the ureter into lower calyx of renal pelvis. VII congeries de la societe internationale d’Urologie.

[5] Hawthorne NJ, Zincke H, Kelalis PP (1976). Ureterocalicostomy: an alternative to nephrectomy. J Urol.

[6] Haouas N, Youssef A, Sahraoui W, Thabet I, Ben Sorba N, Jaidane M (2005). Ureterocalicostomy: indications and results based on a series of 16 patients. Prog Urol.

[7] Virseda JA, Martínez-Ruiz J, Martínez-Sanchiz C, Donate MJ (2011). Ureterocalicostomy: a forgotten surgical technique?. Actas Urol Esp.

[8] Ross JH, Streem SB, Novick AC, Kay R, Montie J (1990). Ureterocalicostomy for reconstruction of complicated pelviureteric junction obstruction. Br J Urol.

[9] Arap MA, Torricelli FC, Mitre AI, Chambo JL, Duarte RJ, Srougi M (2013). Lessons from 90 consecutive laparoscopic dismembered pyeloplasties in a residency program. Scand J Urol.

[10] Shah TP, Vishana K, Joshi RN, Kadam G, Dhawan M (2004). Ureterocalycostomy:A salvage procedure for complex ureteropelvic junction strictures. Indian J Urol.

[11] Jameson SG, Mckinney JS, Rushton JF (1957). Ureterocalyostomy: a new surgical procedure for correction of ureteropelvic stricture associated with an intra-renal pelvis. J Urol.

[12] Couvelaire R, Auvert J, Moulonguet A, Cukier J, leger P (1964). uretero-calicial implantations and anastomoses. Technics and indications. J urol nephrol.

[13] Arap MA, Andrade H, Torricelli FC, Denes FT, Mitre AI, Duarte RJ (2014). Laparoscopic ureterocalicostomy for complicated upper urinary tract obstruction: mid-term follow-up. Int Urol Nephrol.

